# Sclerostin: From Molecule to Clinical Biomarker

**DOI:** 10.3390/ijms23094751

**Published:** 2022-04-26

**Authors:** Ahmed Omran, Diana Atanasova, Filip Landgren, Per Magnusson

**Affiliations:** Department of Clinical Chemistry, and Department of Biomedical and Clinical Sciences, Linköping University, SE-581 85 Linköping, Sweden; ahmed.omran@regionostergotland.se (A.O.); diana.atanasova@regionostergotland.se (D.A.); filip.landgren@regionostergotland.se (F.L.)

**Keywords:** β-catenin, bone, bone formation, immunoassay, LRP6, mechanotransduction, osteocyte, reference interval, *SOST*, Wnt signaling

## Abstract

Sclerostin, a glycoprotein encoded by the *SOST* gene, is mainly produced by mature osteocytes and is a critical regulator of bone formation through its inhibitory effect on Wnt signaling. Osteocytes are differentiated osteoblasts that form a vast and highly complex communication network and orchestrate osteogenesis in response to both mechanical and hormonal cues. The three most commonly described pathways of *SOST* gene regulation are mechanotransduction, Wnt/β-catenin, and steroid signaling. Downregulation of *SOST* and thereby upregulation of local Wnt signaling is required for the osteogenic response to mechanical loading. This review covers recent findings concerning the identification of *SOST*, in vitro regulation of *SOST* gene expression, structural and functional properties of sclerostin, pathophysiology, biological variability, and recent assay developments for measuring circulating sclerostin. The three-dimensional structure of human sclerostin was generated with the AlphaFold Protein Structure Database applying a novel deep learning algorithm based on the amino acid sequence. The functional properties of the 3-loop conformation within the tertiary structure of sclerostin and molecular interaction with low-density lipoprotein receptor-related protein 6 (LRP6) are also reviewed. Second-generation immunoassays for intact/biointact sclerostin have recently been developed, which might overcome some of the reported methodological obstacles. Sclerostin assay standardization would be a long-term objective to overcome some of the problems with assay discrepancies. Besides the use of age- and sex-specific reference intervals for sclerostin, it is also pivotal to use assay-specific reference intervals since available immunoassays vary widely in their methodological characteristics.

## 1. Introduction

In 1637, the island of Urk (now connected to the mainland), in the northern part of the Netherlands, was ravaged by the plague, killing off half of the island’s 300 inhabitants. This event, with its subsequent reduction in the potential number of romantic suitors, would eventually lead to the marriage and inbreeding of a particular Urk-dwelling couple in 1751 [1]. Their descendants, and the distinctive bone disorder they passed along through generations, led van Buchem et al. to the discovery and portrayal of what they named *hyperostosis corticalis generalisata familiaris* in 1955 [2]. A characteristic phenotypic feature was generalized skeletal sclerosis leading to increased bone mineral density typically affecting the cranial base, jaw, and diaphyseal cortex of long bones. The cranial hyperostosis was particularly troublesome since it caused the narrowing of cranial neuroforamina, which subsequently led to facial palsy and deafness while also causing increased intracranial pressure in early adulthood. In 1958, Truswell described a similar disorder found in the Afrikaner community of South Africa, which he named *osteopetrosis with* *syndactyly* [3]. Those affected displayed virtually identical cranial features as in the disease described by van Buchem et al. [2], but these individuals were typically of tall stature and often had signs of syndactyly (fused digits).

In view of the ancestral links between the Dutch and the Afrikaners, a speculation arose that the two conditions might result from mutations in the same gene and that those afflicted might have a common ancestry [4]. In 1998, genome-wide studies were performed on a group of Dutch individuals and their families, which led to the mapping of van Buchem disease to chromosome 17q12-q21 [1]. The mutation has been described as autosomal recessive with loss of function. A year later linkage analysis performed in two inbred families with sclerosteosis proved that the common faulty gene was indeed the same. They further proposed that the possible function of the gene is linked to either inhibition of bone formation or stimulation of bone resorption [1]. The genetic lesions responsible for each disease, however, were shown to have distinct locations, thus disproving the earlier speculation that the two disorders represented a common ancestry.

Two groups of researchers independently identified the *SOST* gene in 2001 [5,6]. This gene was shown to encode a protein with a signal peptide for secretion and a cysteine-knot motif. Loss-of-function mutations in this gene were found in patients with sclerosteosis and thus the physiological role of the *SOST* protein was hypothesized to be suppression of bone formation. Sequencing analysis in two distinctive families from Brazil and USA revealed different mutations in exon 2 that caused a premature stop codon [5,7]. A 52 kb deletion, located 35 kb downstream of *SOST*, was identified as the culprit of van Buchem disease [8]. Later, this deletion was shown to be a noncoding enhancer element, required for the postnatal expression of the *SOST* gene product sclerostin [9].

Although the majority of sclerosteosis cases have been found in the Afrikaner community of South Africa that are descendants of the Dutch settlers from the 17th century, several cases from the USA, Brazil, Germany, Morocco, Turkey, Saudi Arabia, Egypt, Senegal, India, Japan, and China have been reported with 11 different mutations [10]. In addition, two mutations in low-density lipoprotein receptor-related protein 4 (LRP4) have been found in sclerosteosis patients, independent of the sclerostin genotype, demonstrating that LRP4 interacts with sclerostin or mediates its function [11,12,13].

Several other genetic variants have recently been described with various effects on *SOST* expression or gene regulation. The most frequent genetic variant, rs17882143 (*SOST*-p. Val10Ile), located in the signaling peptide, is associated with decreased translocation of sclerostin intra- and extracellularly. It may be associated with a weak decrease in *SOST* expression and the development of non-pathogenic high bone mineral density in the general population [14]. Another variant located in the TATA box motif caused decreased promoter activity [14]. However, information is scarce about the distribution of rare genetic and non-pathological *SOST* variants in the general population and their role in skeletal pathogenesis [15].

## 2. Osteogenesis and Regulation of *SOST* Expression

Osteocytes represent 90–95% of all bone cells in the adult skeleton and are believed to have a lifespan of up to several decades, while osteoclasts live only for a few days and osteoblasts for a few months [16,17,18]. It is believed that the precursors of osteoblasts are skeletal stem cells, formerly known as multipotent mesenchymal stem cells, which also give rise to chondrocytes, muscle cells, and adipocytes [19,20,21].

Bone remodeling is the process by which the body maintains healthy bones and regulates mineral homeostasis. Osteoclasts resorb old bone tissue and osteoblasts form new bone by a mechanism that is strictly regulated by close interaction, known as coupling, of both types of cells. The whole process takes place in small areas of the cortical bone or the trabecular surface, called basic multicellular units, which are formed by osteoclasts, osteoblasts, osteocytes, bone lining cells, and blood vessels [16,22,23]. This complex process of bone remodeling is regulated by several interacting factors including genetic, mechanical (loading, exercise), vascular/nerve, nutritional and by various autocrine, paracrine, and endocrine signals.

Sclerostin was first identified in adult human osteocytes [24], and it has recently been shown that *SOST* is one of the nine most enriched genes in the osteocyte transcriptome signature (*SOST*, *MEPE*, *NGEF*, *WNT1*, *ACKR3*, *TGFB2*, *SEMA3E*, *IRX5*, and *DMP1*) [25]. The strong expression of *SOST* in osteocytes implies a central role in the regulation of bone homeostasis [26]. Although osteocytes are the main cellular source of sclerostin, *SOST* RNA is also expressed in other human and mouse tissues such as cartilage, kidney, heart, liver, epididymis, and vas deferens of the testis, pyloric sphincter, carotid arteries, and parts of the central nervous system [5,27,28]. The biological function of sclerostin outside of the skeleton is less well known. Overexpression of *SOST* in transgenic mice results in reduced osteoblast activity and a lower degree of bone formation resulting in low bone mass and reduced bone strength [26].

*SOST* contains two exons and one intron. The expression is regulated by the non-coding evolutionarily conserved region 5 (ECR5) enhancer site located 35 kb downstream of *SOST* and by a proximal promoter upstream. The transcriptional regulation through either the enhancer or promoter is pathway dependent [29]. The three most commonly described pathways of *SOST* regulation in osteocytes, further discussed in this review, are mechanotransduction [30], Wnt/β-catenin, and steroid signaling [31,32].

### 2.1. SOST Regulation by Mechanotransduction

Mechanical loading is a pivotal factor for bone remodeling since microgravity has a negative impact on bone health in space [33]. In addition, recent research on rodents in space has demonstrated increased osteoclast activity, altered osteoblast differentiation, and osteocyte function [34]. Mice acquired, during a 30-day-long spaceflight, a dramatic increase in *SOST* mRNA expression [35]. Despite the limited research reported (due to practical reasons) on the effect of microgravity on bone health, this pointed to the hypothesis that mechanical loading is an important factor for maintaining a balanced bone remodeling and that this effect could be regulated by sclerostin. Mechanical stimulation by whole-body vibration, a non-pharmacological approach to increase bone mass, was originally developed to prevent loss of bone and muscle mass during prolonged spaceflights [36]. It has also been shown clinically that whole-body vibration results in reduced serum levels of sclerostin, which implies a direct effect on osteocytes through mechanical stimulation [37,38].

The main mechanosensory cell in the skeleton is the osteocyte. Several in vitro and in vivo studies have described molecular pathways in osteocytes that link mechanotransduction to *SOST* regulation [39]. Studying the expression of *SOST* with in vivo models is complex and depends on multiple factors, while the isolation and culturing of primary osteocytes is technically challenging [40]. Therefore, human osteocyte-like cell lines (e.g., MLO-Y4, Ocy 454, and OMGFP66) and/or differentiated osteoblasts and mesenchymal stem cells are mainly used [41,42,43,44,45,46,47].

**Figure 1:**
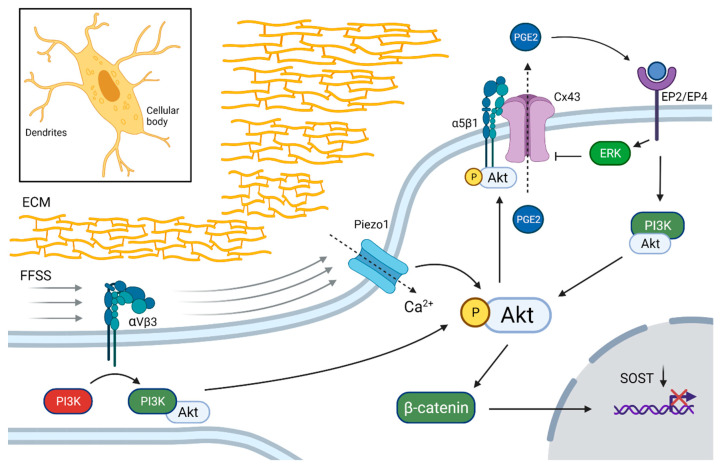
Down-regulation of *SOST* in osteocytes by mechanotransduction. Integrin αVβ3 in dendritic processes sense fluid flow shear stress (FFSS) applied on the extracellular matrix (ECM). The subsequent activation of phosphoinositide 3-kinase (PI3K) phosphorylates Akt, which activates integrin-mediated connexin 43 (Cx43) channel opening on the cellular body. Prostaglandin E2 (PGE2) is transported out of the osteocyte and binds to EP2 and EP4 receptors, which further activates the Akt and extracellular signal-regulated kinase (ERK) pathways and β-catenin, which down-regulates *SOST* expression. FFSS activates also Ca^2+^ influx through Piezo1 channels, which enables enhanced Akt activation. Black dotted arrows indicate transport through membrane channels. Created with BioRender.com, accessed on 15 February 2022.

Low rates of fluid flow shear stress (FFSS) are sensed from the extracellular matrix (ECM) by αVβ3 integrins on the dendrites of osteocytes which, through phosphoinositide 3-kinase (PI3K-Akt), activate the α5β1 integrin-coupled opening of connexin 43 (Cx43)-gap junction channels in osteocytes. Cx43 opening leads to prostaglandin E2 (PGE2) efflux and activation of the PGE2 receptor subtypes EP2 and EP4, which downregulates *SOST* through β-catenin activation [41,48,49]. FFSS also stimulates Ca-channel opening (TRPV4 and Piezzo1) in the cellular body and Ca^2+^ influx, which also activates Akt [42,43,50]. These findings suggest that the Akt pathway is one of the major intracellular mediators of mechanical stimuli in osteocytes that leads to down-regulation of *SOST* (Figure 1).

**Figure 2:**
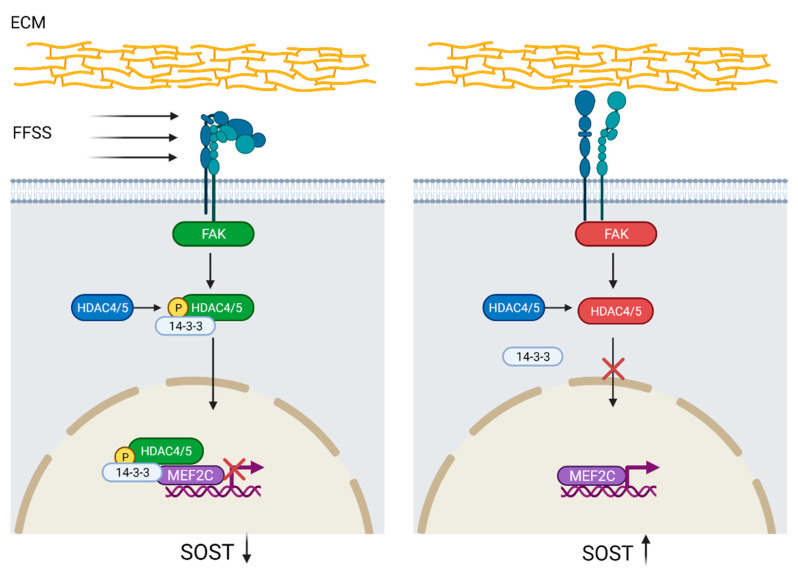
Regulation of *SOST* by histone deacetylase 4/5 (HDAC4/5) activation. Integrin-disintegration, due to fluid flow shear stress (FFSS) in the extracellular matrix (ECM), activates focal adhesion kinase (FAK), which subsequently phosphorylates HDAC4/5. Phosphorylated HDAC4/5 binds to protein 14-3-3 leading to nuclear translocation where, by binding to myocyte-specific enhancer factor 2C (MEF2C) and histone deacetylation at the evolutionarily conserved region 5 (ECR5) site, prevents *SOST* expression. Lack of FFSS precludes HDAC4/5 phosphorylation and thus nuclear translocation, which promotes MEF2C binding to ECR5 and *SOST* upregulation. *SOST* downregulation = black arrow down; *SOST* upregulation = black arrow up. Created with BioRender.com, accessed on 15 February 2022.

Another mechanotransduction pathway has been proposed by Wein et al. [44]. They found that histone deacetylase 4/5 (HDAC4/5) regulates *SOST* expression by histone acetylation at the ECR5 enhancer site, which prevents myocyte-specific enhancer factor 2C (MEF2C) from binding. The transcription factor MEF2C is needed for *SOST* expression and both acetylation at the ECR5 enhancer site and MEF2C knock-down or deletion caused decreased *SOST* expression and also increased bone mass in mice [44,51,52]. In a subsequent study, Sato et al. [45] demonstrated that HDAC4/5 was phosphorylated by focal adhesion kinase and translocated to the nucleus upon FFSS-induced integrin dissociation. Furthermore, lack of FFSS and HDAC4/5 knock-down leads to MEF2C binding at the ECR5 site and increased *SOST* expression, suggesting that phosphorylated HDAC4/5 is a negative regulator of *SOST* (Figure 2) [45,51].

### 2.2. SOST Regulation by Steroid Hormones

**Figure 3:**
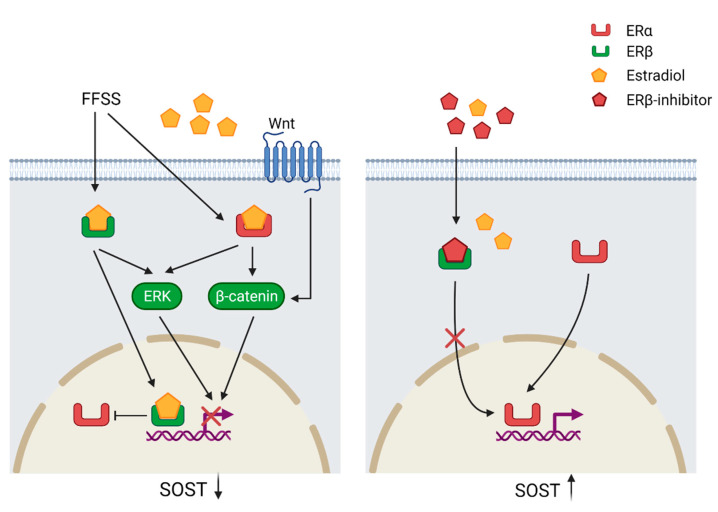
Regulation of *SOST* through the estrogen receptor (ER) pathway. Fluid flow shear stress (FFSS) and estradiol (E2) binding to ERβ suppresses ERα binding to the regulatory motif of *SOST* resulting in *SOST* down-regulation. Estradiol binding to the ERs triggers the extracellular signal-regulated kinase (ERK) and Wnt/β-catenin, which suppress *SOST* expression. The ERα ligand independently maintains basal *SOST* expression and Erβ inhibition prevents down-regulation of *SOST*. *SOST* downregulation = black arrow down; *SOST* upregulation = black arrow up. Created with BioRender.com, accessed on 15 February 2022.

Apart from mechanotransduction, the expression of *SOST* is also regulated by steroid hormone signaling such as estrogen receptors (ER) (Figure 3). The two receptors ERα and ERβ have opposing effects on *SOST* expression. It has been also shown that estradiol downregulates *SOST* expression. While the downregulation by estradiol is mediated by binding to ERβ, the ERα ligand independently maintains *SOST* expression. ERα and ERβ compete for the same *SOST* regulatory site and estradiol enhances the binding of ERβ and thereby prevents ERα from binding to the gene. Loss or inhibition of ERα results also in down-regulation of basal *SOST* expression [53]. Furthermore, treatment with estradiol in human mesenchymal stem cells activates the Wnt/ERα/β-catenin signaling pathway, which results in increased levels of total and active β-catenin [31]. Mechanical strain performed on ulnae from ovariectomized mice resulted in decreased β-catenin activation and increased expression of *SOST* in comparison with sham mice, which suggests that an estrogen/Wnt/β-catenin pathway crosstalk is involved in the regulation of *SOST* [32].

Wijenayaka et al. [54] demonstrated that the steroid hormone 1,25-dihydroxyvitamin D (1,25(OH)_2_D) regulates *SOST* expression. 1,25(OH)_2_D induced expression of *SOST* in osteoblast-like SaOS-2 cells by binding to a vitamin D response element upstream to *SOST* but not by binding to the enhancer site ECR5 [54,55]. Although these findings suggest a direct action of 1,25(OH)_2_D on the regulation of *SOST*, additional studies are needed to elucidate the precise mechanism of action and the regulatory effect in different osteoblast and osteocyte-like cell lines.

## 3. Structural Properties of Human Sclerostin

The product of *SOST*, sclerostin, is a 24 kDa and 213 amino acid-long glycoprotein with a secretion signaling peptide comprising the first 23 amino acids [5]. The circulating form of sclerostin is 22 kDa after cleavage of the signal peptide. Nuclear magnetic resonance analysis shows that the tertiary structure consists of three loops, a cysteine-knot motif with three disulfide bonds, and N- and C-terminal spacer arms [56]. Loops 1 and 3 contain two anti-parallel β-sheets that link with a disulfide bond on the top. Whereas loops 1 and 3 form stable structures, loop 2 is more flexible adopting different stable conformations upon ligand interactions. A structure-based sequence alignment of sclerostin with proteins from the cysteine-knot family reveals a highly conserved cysteine-knot motif, buried in the core of the protein, stabilizing its structure. Most other cysteine-knot proteins form functional homo- or heterodimers. To our knowledge, there is only one experimental study that suggests a possible dimerization or trimerization of sclerostin [57]; however, these findings need to be confirmed in future studies. Moreover, in contrast to the molecular structure of sclerostin, none of the previously identified structural homologs from the cysteine-knot family have long flexible N- and C-terminal arms [56].

**Figure 4:**
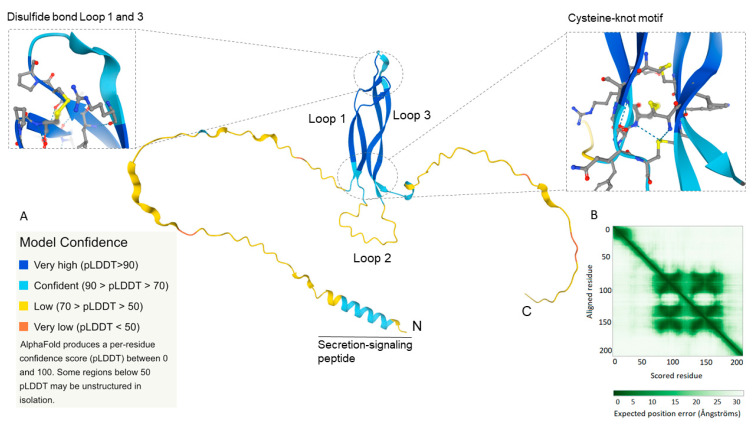
The three-dimensional structure of human sclerostin was generated from the amino acid sequence in the novel AlphaFold Protein Structure Database (https://alphafold.ebi.ac.uk/entry/Q9BQB4) (accessed on 19 September 2021) [58]. The protein comprises a core structure of loops 1 and 3, each having two anti-parallel running β-sheets linking with three disulfide bonds (cysteine-knot motif, magnification to the right; disulfide bond in yellow) and one disulfide bond in the top (magnification to the left; disulfide bond in yellow). Sclerostin contains also a flexible second loop in the bases and two N- and C-terminal spacer arms. (**A**) Confidence score (0–100) of model accuracy based on per-residue predicted local-distance difference test (pLDDT). Loops 1 and 3 have a high to very high confidence score (pLDDT > 70) and loop 2 and the spacer arms low confidence score (pLDDT < 70). (**B**) Expected position error, showing the possible interaction/proximity between the residues, depending on the distance between each residue (in Ångströms). Sclerostin has four closely aligned inner-sequence core domains, each containing one β-sheet.

We performed a computational analysis of the tertiary structure of human sclerostin in the AlphaFold Protein Structure Database (https://alphafold.ebi.ac.uk/entry/Q9BQB4) (accessed on 19 September 2021) and the three-dimensional structure of sclerostin is depicted in Figure 4. AlphaFold is a novel deep learning algorithm developed by DeepMind (London, UK) that performs protein folding predictions based on the amino acid sequence and provides accuracy calculations of the predicted three-dimensional structure [58,59,60]. AlphaFold provides a per-residue confidence measurement from 0 to 100 of the model termed the predicted local-distance difference test (pLDDT). The AlphaFold structure prediction of human sclerostin confirmed the previously described 3-loop conformation with high to very high model confidence (70 > pLDDT > 90; pLDDT > 90; light blue, dark blue) of loop 1 and 3 (Figure 4A). As mentioned, loops 1 and 3 have stable structures and consist of hydrophobic amino acids with the hydrophobic side buried in the core of the loop structure. Additionally, the cysteine-knot motif with three disulfide bonds in close proximity to each other (confidence pLDDT score between 80 and 90) further describes the stable core structure of sclerostin. Furthermore, the predicted aligned error (Figure 4B), that is the expected position error between the aligned residues in Ångströms, is low for the core structure, defining four inner-sequence domains (β-sheets for loops 1 and 3) with high proximity of the residues in the predicted core structure. However, loop 2 and the two spacer arms exhibit a three-dimensional structure with low confidence (70 > pLDDT > 50; pLDDT < 50; yellow, orange) and residues with low proximity. It is worth mentioning that AlphaFold only provides a predicted structure and from the low confidence score for loop 2 and the spacer arms it can be deduced that the three-dimensional orientation, proximity, or correct folding, would circumstantially vary depending on different physicochemical conditions and molecular interactions.

**Figure 5:**
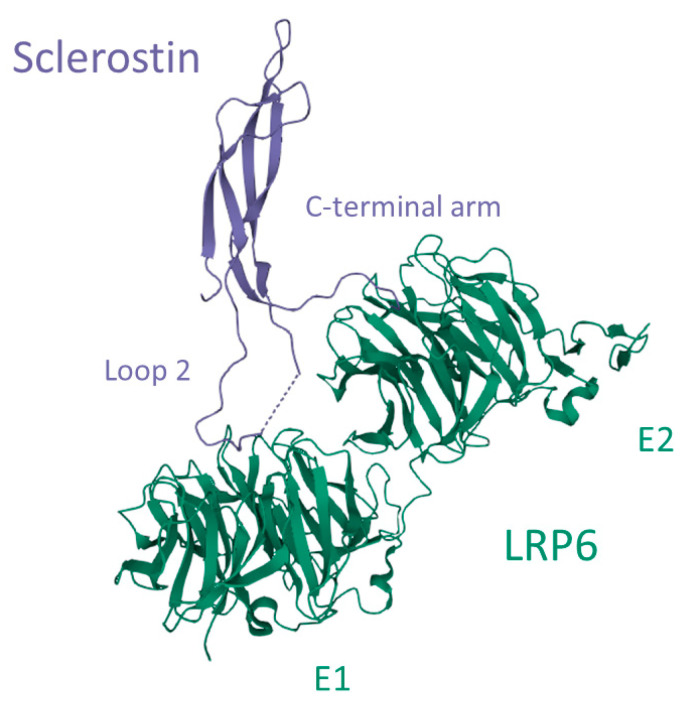
Interaction between sclerostin and the low-density lipoprotein receptor-related protein 6 (LRP6). Loop 2 of sclerostin binds to the E1 ectodomain of LRP6, while a part of the C-terminal arm interacts with the E2 ectodomain. Image created in the Research Collaboratory for Structural Bioinformatics—Protein Data Bank with the Mol* Viewer [64], based on the crystal structure LRP6 E1-E2-*SOST* complex, PDB-ID: 6L6R [63].

Sclerostin interacts with glycosaminoglycans such as heparin/heparan sulfate by electrostatic interactions between arginine and lysine side chains on the hydrophilic surface and sulfate groups of heparin. This molecular interaction mediates the transportation of sclerostin to the cell surface and embedding in the extracellular matrix [56]. Heparan sulfate is produced by bone cells and is a component of the extracellular matrix. It also facilitates the excretion of soluble proteins from the cells. Sclerostin interacts preferably with full-length heparin and this interaction is enhanced by sulfation and high divalent cation concentrations such as Ca^2+^ and Zn^2+^ [61]. Sclerostin interacts also with the co-receptors LRP4, 5, and 6 and thereby impedes the binding of mainly Wnt1 but also Wnt3a. It has been reported that sclerostin is a more potent inhibitor of Wnt1 in comparison with Wnt3a and also that the binding mechanism of sclerostin to the Wnt1 and Wnt3a receptors differs [62]. Through crystal structure analysis, Kim and colleagues [63] proposed an interactive mechanism of sclerostin with LRP6 (Figure 5). LRP6 is a transmembrane protein that consists of four identical ectodomains (E1, E2, E3, and E4), and the co-receptor interaction of LRP6 with the G-protein coupled receptor Frizzled depends on the type of ligand binding. While Wnt3a interacts with E3 and E4, and another Wnt-inhibitor dickkopf-1 (DKK1) interacts with the E1 and E4 ectodomains, both Wnt1 and sclerostin interact with the E1 and E2 ectodomains of LRP6 [62]. Sclerostin interacts with E1 and E2 by binding with loop 2 and partially with the C-terminal arm, respectively (Figure 5) [63]. This interaction inhibits directly the binding of Wnt1 and allosterically prevents the binding of Wnt3a to LRP6 and thereby prevents receptor-coupling with Frizzled on osteoblasts [62]. This inhibition leads to extracellular events that will be further discussed in this review.

## 4. Functional Properties of Sclerostin

**Figure 6:**
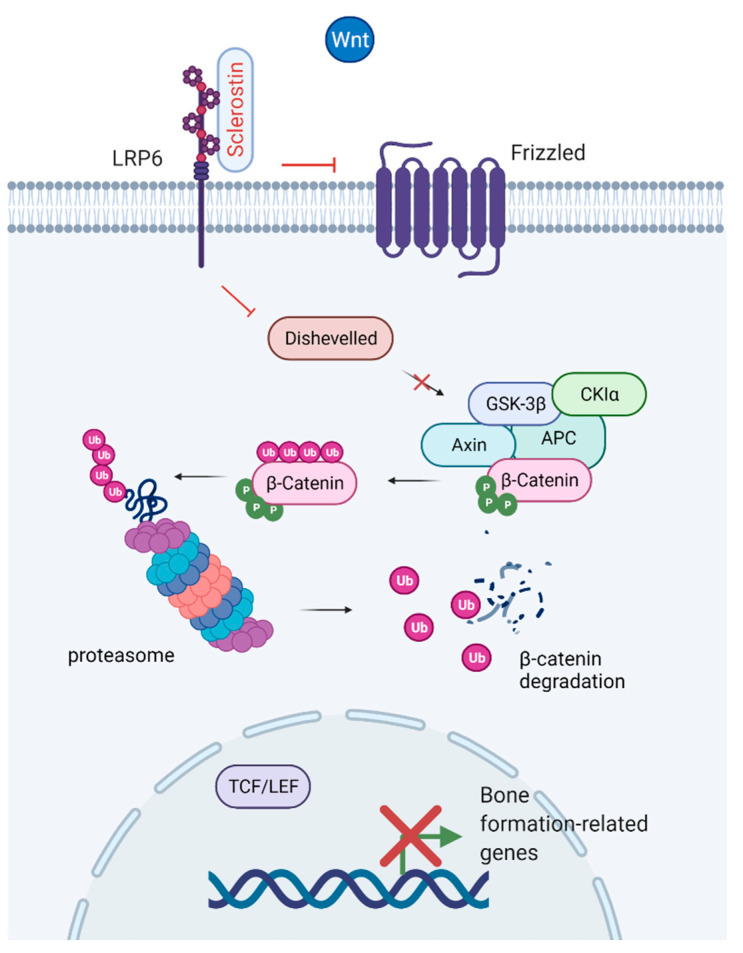
Inhibition of the Wnt/β-catenin canonical pathway. Binding of sclerostin to LRP6 prevents coupling with the Frizzled receptor. Deactivation of the receptor results in phosphorylation of β-catenin by the APC-GSK3β complex, translocation of β-catenin to the proteasome and degradation. The enhanced degradation of β-catenin prevents nuclear translocation and binding to the T-cell factor/lymphoid enhancer factor (TCF/LEF) transcription factor complex, which leads to reduced expression of genes related to bone formation. Created with BioRender.com, accessed on 15 February 2022.

The Wnt signaling pathway is a critical regulator of the development and maintenance of tissues, including bone, working through the stimulation of Runx2 gene expression [65,66]. The discovery of the Wnt/β-catenin signaling system in bone led to an increased interest in Wnt signaling within the bone research community [67,68]. Canonical Wnt signaling promotes osteogenesis through its effects on mesenchymal stem cells by promoting osteoblastic lineage whilst inhibiting osteoclast, adipocyte, and chondrocyte differentiation [67,69,70]. Binding of Wnt to its receptor complex, LRP5/6, and the Frizzled receptor, facilitates β-catenin stabilization and accumulation in the cytoplasm. β-catenin is translocated to the nucleus, where it binds to T-cell factor/lymphoid enhancer factor (TCF/LEF), causing displacement of transcriptional corepressors, and initiates transcription of Wnt target genes [71]. Sclerostin is a negative regulator of Wnt signaling [24,37]. By binding to LPR4 and LPR5/6, sclerostin inhibits canonical Wnt signaling leading to phosphorylation of β-catenin and thereby enhances its degradation by proteasomes (Figure 6) [72]. DKK1 is another endogenously secreted factor that acts as an inhibitor of the Wnt pathway through the Wnt co-receptor LPR5/6 [73]. A potential role in Wnt signaling has also been suggested for fibroblast growth factor 23 (a regulator of phosphate metabolism secreted by osteoblasts and osteocytes). Mäkitie et al. [74] found normal levels of sclerostin and DKK1 but increased levels of fibroblast growth factor 23 in *WNT1* mutation-positive individuals with impaired Wnt signaling and early-onset osteoporosis.

## 5. Pathophysiology and Biological Variability of Sclerostin

Inheritance, environmental factors, age, sex, body mass index, and total body fat content all influence the circulating levels of sclerostin. Declining bone mass with age is associated with increased expression of sclerostin in osteocytes with a subsequent reduction in Wnt/β-catenin signaling in bone cells [75,76,77]. Unlike the bone resorption marker C-terminal telopeptide of type I collagen (CTX) and the bone formation marker osteocalcin, serum sclerostin is unaffected by the time of blood sampling [78,79].

Different pathological disorders may influence the expression of *SOST* and subsequently the circulating levels of sclerostin. Paget’s disease of bone (PDB) is a focal chronic metabolic disorder affecting mostly elderly individuals. PDB is characterized by unbalanced bone remodeling resulting in areas of increased bone resorption and other areas with abnormal formation of new bone, with the primary cellular abnormality in osteoclasts [80]. Elevated sclerostin levels were observed in older patients with PDB although they did not correlate with other markers of bone turnover [81]. Yavropoulou et al. showed that circulating sclerostin levels are significantly increased in patients with increased bone turnover, regardless of underlying pathologies such as PDB [82]. Another study did not find any differences in sclerostin levels between patients with active or inactive PDB [83].

Patients with chronic kidney disease have higher levels of sclerostin in comparison with healthy individuals and serum sclerostin tends to increase as the kidney function declines. In reported mice models, an early increase in sclerostin-positive osteocytes correlated with an increased expression of inactive phosphorylated β-catenin, which suggests an overall repression in Wnt/β-catenin signaling that may contribute to the progression of chronic kidney disease–mineral bone disorder (a.k.a. CKD–MBD) [84,85]. In humans, sclerostin was reported to be positively correlated with age and to be higher in men than women even in patients with chronic kidney disease [86,87,88,89]. Boltenstål and colleagues [90] studied a cohort of patients with chronic kidney disease stages 3–5D and showed that sclerostin levels were higher in low bone turnover patients in comparison with non-low bone turnover patients. A recent study by Nakagawa and colleagues on 654 hemodialysis patients showed that higher levels of sclerostin were associated with lower levels of parathyroid hormone and bone-specific alkaline phosphatase [91]. However, these associations were substantially attenuated when the results were statistically adjusted for parathyroid hormone, which suggests that sclerostin is not the main mediator driving the effects of parathyroid hormone on bone remodeling.

Diabetes is associated with alterations in bone mineral density, growth, and bone remodeling, with an increased risk of fractures [92,93,94]. Bone-derived factors including osteocalcin, osteoprotegerin, and sclerostin may be altered because of disruption of the glucose metabolism [95], and different mechanisms have been suggested in the pathogenesis of diabetes-associated skeletal disease [96]. A recent review anticipated the possible interaction between bone and fat tissue in the regulation of glucose metabolism [97]. Sclerostin was negatively associated with C-peptide and HbA1c in children with type 1 diabetes implying an interaction between sclerostin and glucose metabolism [98]. Elevated levels of sclerostin, independent of age and sex, have also been shown in patients with type 2 diabetes [99,100,101].

### Sclerostin as a Treatment Target

Osteoporosis is a global health problem and the most common chronic metabolic disease with a higher incidence in Caucasians, older individuals, and women. The European Foundation for Osteoporosis and Bone Disease defined osteoporosis as a progressive systemic skeletal disease characterized by low bone mass and microarchitectural deterioration of bone tissue, with a consequent increase in bone fragility and susceptibility to fracture [102]. Biochemical markers of bone turnover are clinically useful for monitoring the treatment of osteoporosis, although they cannot be used for the diagnosis of osteoporosis [103].

Sclerostin has been considered a target protein to be neutralized with IgG2 monoclonal antibodies, known as romosozumab, originally AMG785, (Amgen Inc., Thousand Oaks, CA, USA; and UCB Pharma, Brussels, Belgium), which activates Wnt signaling, thereby increasing bone formation, inhibiting bone resorption, and increasing bone mass in patients with osteoporosis [104]. Considerable interest has emerged in this anti-sclerostin antibody ever since the first clinical trial was reported in 2011 by Padhi et al. [105]. Two major phase III trials, by Cosman et al. [106] and Saag et al. [107], demonstrated that treatment with romosozumab decreased the incidence of fractures in patients with osteoporosis. Both studies proposed romosozumab as a promising therapy in patients with osteoporosis [108]. These extensive trials and expanded evidence of the beneficial effects of anti-sclerostin treatment led to its approval in the US, EU, Canada, and Japan in 2019. Treatment with romosozumab has also been suggested to have a positive effect on bone diseases associated with multiple myeloma [109]. Patients with plasma cell malignancies such as multiple myeloma have elevated levels of sclerostin, which may contribute to the pathophysiology of lytic bone lesions associated with this hematological malignancy [110,111].

## 6. Current Assays—Sclerostin and Bioactive Sclerostin

Although not clinically proven, as of today, the assessment of circulating sclerostin may have a potential role in the routine clinical setting for different disorders affecting bone remodeling and for monitoring patients treated with different therapeutic modalities. A large number of assays are available (for research use) for the measurement of circulating (Table 1). However, there is little knowledge about how results from different assays are concordant with each other, which confounds direct comparisons between different clinical trials applying various sclerostin assays. Piec et al. [112] revealed significant variability in three different sclerostin assays arguing the accuracy and specificity of these assays. Hitherto, only a few assays have been developed to measure the bioactive/intact sclerostin (i.e., Biomedica (Vienna, Austria), DiaSorin Inc. (Stillwater, MN, USA), and TECOmedical (Sissach, Switzerland)).

Most of the available sclerostin tests are provided as enzyme-linked immunosorbent assays (ELISA) that notably differ regarding assay range, sensitivity, and turnaround time (Table 1). Except for a few companies, most manufacturers do not provide specific information regarding the traceability of the standards used in their assays. As shown in Table 1, two ELISA kits from different manufacturers, i.e., G-Biosciences (St. Louis, MO, USA) and LifeSpan BioSciences (Seattle, WA, USA) have exactly the same assay range, which indicates that they may have identical standards from the same manufacturer but referred to different assay brand names; however, we did not confirm this by contacting the manufacturers. An alternative to ELISA is provided by Meso Scale Discovery (Rockville, MD, USA) implementing multiplex analysis (Human Bone Panel I Kit) in which sclerostin is included besides two other bone biomarkers (i.e., osteoprotegerin and alkaline phosphatase).

To our knowledge, the LIAISON^®^ sclerostin (DiaSorin Inc.) chemiluminescence immunoassay (CLIA) is the only fully automated method available. This assay is designed to detect only intact sclerostin and has the shortest turnaround time (1 h) of all assays. Drake et al. [113] compared the LIAISON^®^ sclerostin assay with two other methods, namely Biomedica sclerostin and Meso Scale Discovery (Human Bone Panel I Kit), which demonstrated that the LIAISON^®^ sclerostin CLIA has an enhanced sensitivity and matched plasma and serum equivalence. It should, however, be noted that Biomedica recently has developed and released an assay for bioactive (intact) sclerostin that might be comparable with the intact sclerostin LIAISON^®^ assay.

Another study by Durosier et al. [114] on 189 healthy adults showed that sclerostin levels were markedly different when measured by three different assays, namely Meso Scale Discovery, Biomedica, and TECOmedical. The Meso Scale Discovery assay resulted in low absolute values, which the authors suggested could be due to this assay specifically detecting intact sclerostin, while the two other assays detect circulating fragments of sclerostin [114,115]. However, the antibody specificity for the Meso Scale Discovery assay towards intact sclerostin has been questioned by others and, importantly, the assay protocol does not state that this method is specific for intact sclerostin [113].

Taken together, the value of assessing sclerostin in the routine clinical setting has yet to be shown and described discrepancies between assays will influence the use of sclerostin in clinical practice. Reported immunoassay variabilities for the measurement of sclerostin increase the need for further comparative studies that may improve the understanding of sclerostin in clinical studies. Although not investigated, it is likely that results from different sclerostin assays cannot be compared with each other even if normalized against healthy individuals. A long-term objective to overcome problems with discrepancies between sclerostin values generated by different assays would be to design projects working on assay harmonization or standardization. The International Osteoporosis Foundation (IOF) and the International Federation of Clinical Chemistry and Laboratory Medicine (IFCC) have established a working group, i.e., the IOF-IFCC Joint Working Group on Bone Marker Standards (a.k.a. WG-BMS). This working group has performed significant work towards the harmonization of the bone markers type I procollagen intact N-terminal propeptide (a.k.a. PINP) assays and the standardization of CTX assays in collaboration with the assay manufacturing industry [116], and would thus be the appropriate forum for a project regarding sclerostin assays.

### Sclerostin Reference Intervals

Reported reference intervals for serum and plasma sclerostin in adult individuals are presented in Table 2. Besides peer-reviewed articles, Table 2 also includes information about reference intervals given by different companies in the instructions for use. The circulating levels of sclerostin are associated with age and sex where males, in general, have higher sclerostin levels than females across the adult lifespan. These sex-specific differences are also noted during childhood and adolescence, particularly during early puberty where boys have higher sclerostin levels in comparison with girls and, later on, serum sclerostin levels decline in late puberty in both sexes [117]. It is therefore essential to use age- and sex-specific reference intervals for the interpretation of sclerostin in all age groups. Osteogenesis differs in children and adolescents from that of adult individuals since it, besides remodeling, also covers modeling and linear growth of the skeleton. These developmental differences are reflected by biomarkers of bone metabolism and it has been widely reported that children, and in particular adolescents during the pubertal growth spurt, have higher circulating levels of biochemical bone markers of bone turnover in comparison with adults [118]. Grouping biochemical tests by means of Tanner stage partitioning is a complementary and alternative approach to age- and sex-specific reference intervals that can be applied because it reflects bone growth and development that influence biomarkers of bone metabolism [119]. Reflecting the methodological characteristics and reported discrepancies between assays (described above), it is also pivotal to use assay-specific reference intervals when evaluating and comparing measured sclerostin values.

Most sclerostin assays have validated reference intervals for both serum and plasma samples, and it should be observed that measurements of sclerostin are consistently higher in plasma in comparison with serum samples (Table 2). The reason for this observation is currently unknown, but it was initially suggested that this was due to molecular interactions between heparin and reported heparin-binding sites on sclerostin, which potentially could interfere with sclerostin binding proteins by exposing recognizable epitopes for assay antibodies towards sclerostin [56]. However, later studies on EDTA and citrate plasma revealed that blood samples collected with these additives also have higher levels of sclerostin in comparison with serum samples (Table 2) [120].

**Table 2:** Reported reference intervals for serum and plasma sclerostin in adult individuals.

Reference/Source (Year)	Assays	Reference Interval ^a^ (pmol/L)
Mödder et al. [75] (2011)	Biomedica	Healthy subjects, 21–97 years Pre-menopausal females, n = 123: 24 ± 1 ^b^ Post-menopausal females, n = 152: 28 ± 1 Males, n = 318: 33 ± 1
McNulty et al. [121] (2011)	Biomedica	Healthy subjects, 20–59 years, n = 25 Serum: 44 ± 5; heparin plasma: 65 ± 6 ^b^
Ardawi et al. [122] (2011)	Biomedica	Pre-menopausal females, n = 1235: 7.5–46 Post-menopausal females, n = 568: 23–74
Durosier et al. [114] (2013)	Biomedica Meso Scale Discovery	Healthy subjects, 65 ± 1 years, n = 187 Biomedica: 20–101 Meso Scale Discovery: 0.79–3.0
Piec et al. [112] (2016)	Biomedica TECOmedical, High sensitive R&D Systems	Healthy subjects, 18–26 years, n = 46 Biomedica: 22–59 (EDTA plasma: 23–71) ^c^ TECOmedical: 11–33 (EDTA plasma: 14–49) R&D Systems: 2.7–13 (EDTA plasma: 15–53)
Drake et al. [113] (2018)	LIAISON^®^ Sclerostin	Healthy subjects, 21–97 years Females, n = 265: 6.2–36 Males, n = 271: 8.2–51
Kerschan-Schindl et al. [123] (2022)	Biomedica (Bioactive sclerostin)	Healthy subjects, 26–74 years Females, n = 175: 51 (38–70) ^d^ Males, n = 61: 62 (41–92)
Biomedica (instructions for use)	Sclerostin, BI-20492	Healthy subjects, n = 411: 11–52
Biomedica (instructions for use)	Bioactive sclerostin BI-20472	Healthy subjects Serum, n = 32, 12–143 EDTA plasma, n = 24, 29–226
R&D Systems (Instructions for use)	Quantikine, DSST00	Healthy subjects, n = 35 Serum: 2.9–13 EDTA plasma: 8.8–31 Heparin plasma: 9.8–35
TECOmedical (Instructions for use)	TECO^®^ High sensitive	Pre-menopausal females, n = 20: 9–33 Post-menopausal females, n = 19: 11–40 Males, n = 10: 25–51

^a^ Reference intervals given for serum samples if not stated otherwise. ^b^ Mean ± SEM. ^c^ Minimum–maximum. ^d^ Median (interquartile range). Reference intervals presented as the mean ± 2SD, or 95% confidence intervals, if not stated otherwise. All sclerostin values are expressed in pmol/L using the conversion factor of 0.044 from pg/mL to pmol/L, i.e., 1 pg/mL = 0.044 pmol/L. Note that reference intervals for discontinued assays are excluded even if they are reported in the given references.

## 7. Summary and Conclusions

This narrative review covers the in vitro regulation of human sclerostin and *SOST* gene expression, structural and functional properties of sclerostin, pathophysiology, biological variability, and recent assay developments for measuring circulating sclerostin. By employing the novel AlphaFold Protein Structure Database, with a deep learning algorithm voted Method-of-the-Year 2021 in *Nature Methods* [60], we generated a comprehensive, state-of-the-art three-dimensional structure prediction for human sclerostin. Reported methodological discrepancies and inconsistencies among immunoassays for the measurement of sclerostin increase the need for further comparative studies that may improve the understanding of sclerostin in clinical studies. The assessment of circulating sclerostin may have a potential role in the routine clinical setting; however, this has yet to be proven and clinically validated in different disorders affecting bone and mineral metabolism.

## Figures and Tables

**Table 1:** Commercially available assays for determination of circulating human sclerostin.

Company	Assay Name	Assay Range (pmol/L)	Sensitivity (pmol/L)
Biomedica	Sclerostin ELISA	15–240	3.2
Biomedica	Bioactive Sclerostin ELISA	10–320	1.9
Boster Bio	Sclerostin/*SOST* ELISA PicoKine™	1.4–88	<0.4
G-Biosciences	Immunotag™ *SOST* (Sclerostin)	2.8–176	1.7
LifeSpan BioSciences	*SOST*/Sclerostin ELISA	2.8–176	1.7
R&D Systems	*SOST*/Sclerostin Quantikine ELISA	1.4–88	0.17
RayBiotech	RayBio^®^ *SOST* ELISA	1.8–440	1.8
TECOmedical	Sclerostin TECO^®^ High sensitive	2.2–132	0.44
Invitrogen	Sclerostin (SOST) ELISA	1.8–440	0.66
**Assays other than ELISA**			
DiaSorin	LIAISON^®^ Sclerostin, by automated CLIA	2.2–264	0.88
Meso Scale Discovery	Human Bone Panel I Kit, by Multiplex	0–440	0.05

All sclerostin values are expressed in pmol/L using the conversion factor of 0.044 from pg/mL to pmol/L, i.e., 1 pg/mL = 0.044 pmol/L. Abbreviations: CLIA, chemiluminescence immunoassay; ELISA, enzyme-linked immunosorbent assay.

## Abbreviations

1,25(OH)_2_D	1,25-dihydroxyvitamin D
CLIA	Chemiluminescence immunoassay
CTX	C-terminal telopeptide of type I collagen
Cx43	Connexin 43
DKK1	Dickkopf-1
ECM	Extracellular matrix
ECR5	Evolutionarily conserved region 5
ELISA	Enzyme-linked immunosorbent assay
EP2	Prostaglandin E2 receptor subtype 2
EP4	Prostaglandin E2 receptor subtype 4
ER	Estrogen receptor
ERK	Extracellular signal-regulated kinase
FAK	Focal adhesion kinase
FFSS	Fluid flow shear stress
HDAC4/5	Histone deacetylase 4/5
LRP	Low-density lipoprotein receptor-related protein
MEF2C	Myocyte-specific enhancer factor 2C
PDB	Paget’s disease of bone
PGE2	Prostaglandin E2
PI3K	Phosphoinositide 3-kinase
pLDDT	Predicted local-distance difference test
TCF/LEF	T-cell factor/lymphoid enhancer factor
Wnt	Wingless-related integration site
